# Human Urine-Derived Stem Cells: Potential for Cell-Based Therapy of Cartilage Defects

**DOI:** 10.1155/2018/4686259

**Published:** 2018-04-17

**Authors:** Long Chen, Lang Li, Fei Xing, Jing Peng, Kun Peng, Yuanzheng Wang, Zhou Xiang

**Affiliations:** ^1^Department of Orthopedics, West China Hospital, Sichuan University, Chengdu, Sichuan, China; ^2^Department of Orthopedics, Guizhou Provincial People's Hospital, Guiyang, Guizhou, China; ^3^Department of Orthopedics, The Second Affiliated Hospital of Nanchang University, Nanchang, China

## Abstract

Stem cell therapy is considered an optimistic approach to replace current treatments for cartilage defects. Recently, human urine-derived stem cells (hUSCs), which are isolated from the urine, are studied as a promising candidate for many tissue engineering therapies due to their multipotency and sufficient proliferation activities. However, it has not yet been reported whether hUSCs can be employed in cartilage defects. In this study, we revealed that induced hUSCs expressed chondrogenic-related proteins, including aggrecan and collagen II, and their gene expression levels were upregulated *in vitro*. Moreover, we combined hUSCs with hyaluronic acid (HA) and injected hUSCs-HA into a rabbit knee joint with cartilage defect. Twelve weeks after the injection, the histologic analyses (HE, toluidine blue, and Masson trichrome staining), immunohistochemistry (aggrecan and collagen II), and histologic grade of the sample indicated that hUSCs-HA could stimulate much more neocartilage formation compared with hUSCs alone, pure HA, and saline, which only induced the modest cartilage regeneration. In this study, we demonstrated that hUSCs could be a potential cell source for stem cell therapies to treat cartilage-related defects in the future.

## 1. Introduction

Cartilage defects caused by trauma injury and osteoarthritis (OA) are a major public health threat worldwide [1]. Cartilage defects lead to the restriction of joint activities, resulting in pain and a poor quality of life. Currently, treatment options for cartilage defects include physiotherapy, external medication, intra-articular irrigation, chondroplasty, microfracture, and mosaicplasty. However, these treatments cannot consistently stimulate the production of hyaline cartilage for tissue repair, completely fill the empty of the defect, or integrate repaired tissue with adjacent native tissue [2]. To address the issues, tissue engineering is considered to be a promising alternative strategy for the regeneration of cartilage.

Autologous chondrocyte implantation (ACI) is a type of cell therapy, in which healthy chondrocytes are harvested from nonlesion areas and transplanted back into lesion areas [3]. Recently, the Food and Drug Administration (FDA) in the United States approved the usage of autologous chondrocytes cultured on porcine collagen membrane (Maci) for repairing full-thickness cartilage defects in adult patients. For both ACI and Maci procedures, it is technically challenging to obtain a high density of chondrocytes and maintain their differentiation state [3–5]. Therefore, other cell sources need to be explored for tissue engineering. Previous studies demonstrated that mesenchymal stem cells (MSCs), such as human adipose tissue-derived stem cells (hASCs) and human bone marrow mesenchymal stem cells (hBMSCs), were potential stem cell sources for the applications of cartilage tissue engineering approaches [6, 7]. However, the sources of hBMSCs are limited and the procedure of obtaining hASCs is invasive, which urges the demand for more practical and suitable cell sources for tissue engineering of cartilage.

Since Zhang et al. initially described the isolation of MSCs from the human urine [8], hUSCs have received significant attention, and several advantages of hUSCs have been identified. Firstly, hUSCs show robust proliferation ability and have the capacity for multipotent differentiation [9]. Secondly, hUSCs can be accessed via a simple, noninvasive, and low-cost approach, and thus surgical procedures are avoided [10]. More importantly, hUSCs that are isolated from autologous urine do not induce immune responses or rejection. In addition, since no invasive and painful procedures are involved during urine collection, there are fewer ethical issues. In previous studies [9, 11, 12], hUSCs were shown to differentiate into neuron-like cells, urothelial cells, smooth muscle cells, and osteoblasts, which have been successfully applied in studies involving neural, urinary, and bone tissue regeneration [9, 11, 12]. However, it has not yet been reported whether hUSCs can be applied to tissue regeneration of cartilage.

A novel strategy for the regeneration of cartilage defects involves seeding cells into/onto biomaterials [13]. Biomaterials provide a suitable microenvironment for cells, including mechanical support for engineered tissues [14]. Hyaluronic acid (HA) is an important component of synovial fluid, which protects joint cartilage by lubricating and absorbing shock [15]. Hence, HA is able to offer a suitable platform for cartilage repairing and is commonly used.

Therefore, in this study, we obtained hUSCs according to the previously described procedure of isolation and culture [8, 10] and assessed their capacity for chondrogenesis. We also investigated whether hUSCs could serve as a potential cell source for cartilage tissue engineering via comparing the therapeutic effects of hUSCs plus HA, hUSCs alone, HA alone, and normal saline after injections into cartilage defects of a rabbit knee joint. The therapeutic outcome was evaluated by gross appearance and histological and immunohistochemical analyses.

## 2. Materials and Methods

This study was conducted under the Guide for the Care and Use of Laboratory Animals of the National Institutes of Health. The Research Ethics Board for both human samples and animal protocols was approved by the Ethics Committee of West China Hospital, Sichuan University, Chengdu, China.

### 2.1. Isolation and Proliferation of Human Urine-Derived Stem Cells

Primary hUSCs were obtained from five healthy male adult donors, who were between 23 and 27 years old (mean age: 25 years) without urinary system disease, using the methods that were described previously [8, 10]. A total of 200 mL of the sterile urine sample was collected from each person, and subsequent steps were performed separately. Each sample was added with 1% penicillin and streptomycin and centrifuged for 10 minutes at 1500 rpm. The cell pellet was resuspended in 25 mL of phosphate-buffered saline (PBS) and centrifuged again for 10 minutes at 1500 rpm. Then, cells were seeded in 24-well plates with culture medium comprised of keratinocyte serum-free medium (KSFM) and embryonic fibroblast medium (EFM) at a ratio of 1 : 1 as well as 5% fetal calf serum (FBS) [8, 10]. The medium was changed every three days, and cells were passaged by using trypsin after reaching subconfluency.

To evaluate cell proliferation, hUSCs were seeded into a 96-well plate and incubated in 100 *μ*L of cell culture medium at 37°C and 5% CO_2_. Cell viability was assessed at days 0, 1, 3, 5, 7, and 9 using the Cell Counting Kit-8 (CCK-8; Life Technologies, USA). At each time point, 10 *μ*L of CCK-8 reagent was added to each well and the optical density was measured using a spectrophotometer at a wavelength of 490 nm with a background correction at 630 nm.

### 2.2. Flow Cytometry Analysis

When at passage 4 (P4), hUSCs were harvested using trypsin-EDTA, and 1 × 10^6^ hUSCs were resuspended in Hank's Balance Salt Solution (HBSS) supplemented with 1% (*v*/*v*) bovine serum albumin (BSA). Cells were incubated for 30 min at 4°C in the dark and followed by the following monoclonal antibodies: CD34-APC (BD, USA), CD45-PE (BD, USA), HLA-DR-PE (BD, USA), CD29-PE (BD, USA), CD73-PE (BD, USA), CD90-FITC (BD, USA), CD105 (Abcam, UK), and CD166 (Abcam, UK). Next, cells were washed with PBS and incubated with the appropriate secondary antibodies. Cells were analyzed using the Beckman Cytomics FC500 Flow Cytometry Analyzer (Beckman Coulter, USA).

### 2.3. Multilineage Differentiation Potential of Human Urine-Derived Stem Cells

#### 2.3.1. Osteogenic Induction

To induce osteogenic differentiation, hUSCs were cultured at a density of 5 × 10^3^ cells/well in a 6-well plate for 21 days under the condition of 37°C and 5% CO_2_ in osteogenic medium (Cyagen Biosciences Inc., USA). The medium was replaced every 3 days. After induction, cells were fixed with 75% ethanol for 20 min and stained with Alizarin red solution (Sigma, USA) for 30 min.

#### 2.3.2. Adipogenic Induction

hUSCs (4th passage) were cultured at a density of 5 × 10^3^ cells/well in a 6-well plate and induced using adipogenic medium (Cyagen Biosciences Inc., USA). The medium was replaced every 3 days. After a total of 14 days, cells were fixed using 10% formalin and stained with Oil red O solution (Sigma, USA) for 30 min to visualize lipid vacuoles.

#### 2.3.3. Chondrogenic Differentiation

To induce chondrogenic differentiation, 1 × 10^6^ hUSCs were centrifuged for 5 min at 1500 rpm after which the pellet was resuspended in chondrogenic medium (Cyagen Biosciences Inc., USA). After the 21-day induction, toluidine blue solution was used to visualize extracellular matrix-bound proteoglycans. For immunofluorescence purposes, the pellet was embedding in optimum cutting temperature (OTC) compound after chondrogenic differentiation. Cells were subsequently incubated with anti-type II collagen (1 : 100; Novus, USA) and anti-aggrecan (1 : 100; Novus, USA) antibodies for 2 h, washed twice in PBS, and stained with anti-mouse Alexa Fluor 594 IgG (1 : 200; Jackson, USA). Nuclei were stained with DAPI. Cells were observed using a fluorescence microscope (Olympus IX50, Japan).

### 2.4. Quantitative Reverse Transcriptase Polymerase Chain Reaction (RT-PCR)

After chondrogenic induction for 21 days, cellular RNA was extracted using TRIzol reagent (Life Technologies, USA) and reverse-transcribed into cDNA using a PrimeScript RT reagent Kit (Takara, Japan). The expression of specific genes was quantified using an SYBR Premix Ex Taq II kit (Takara, Japan) in an IQ5 real-time system (Bio-Rad, USA). Primer sequences used for qPCR are presented in Table 1. The target gene expression was analyzed and compared to h-actin, which served as a reference gene.

### 2.5. Human Urine-Derived Stem Cells for Cartilage Tissue Engineering: *In Vivo* Study

#### 2.5.1. Establishing the Mixture of hUSCs-Hyaluronic Acid for Injection

Hyaluronic acid (HA) was purchased from Furuida Biosciences (the concentration of HA solution was 1%). hUSCs and HA were mixed at a ratio of 1 × 10^7^ : 1 mL. The mixture was supplied with the hUSC culture medium.

#### 2.5.2. Cell Morphology, Viability, and Proliferation in hUSCs-HA

Cell morphology of hUSCs in HA was compared with that of hUSCs in PBS to assess the cell state. The viability of the cells in HA was determined using an annexin V-FITC/PI apoptosis detection kit [16]. Twenty-four hours after establishing the hUSCs-HA solution system, hUSCs were retrieved from the hUSCs-HA solution by centrifugation. The cell suspension was stained with 5 *μ*L of FITC-conjugated annexin V and 10 *μ*L of PI. Cells were analyzed by FACS Calibur (BD, USA).

Cell proliferation of hUSCs-HA and hUSCs-PBS was assessed at days 0, 1, 3, 5, and 7 using a CCK-8 kit (Life Technologies, USA) in the hUSC culture medium with HA or PBS. At each time point, 10 *μ*L of CCK-8 reagent was added to each well, and the optical density was measured using a spectrophotometer at a wavelength of 490 nm with a background correction at 630 nm.

#### 2.5.3. Animal Model

All surgeries were performed under general sodium pentobarbital anesthesia, and efforts were made to minimize the suffering of the animals. In this study, a total of twenty-four 12-week-old New Zealand white rabbits (2–2.5 kg, no gender limitation) were purchased from Chengdu Dashuo Laboratory Animal Limited Company. All the surgeries were conducted on both sides.

**Figure 1:**
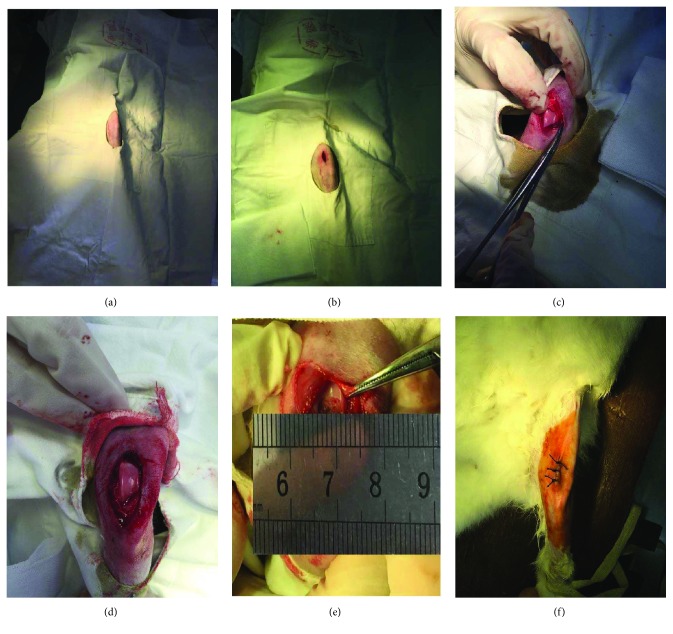
The process of the establishment of cartilage defect model. (a) General disinfection of the knee. (b) An incision was made on the medial joint of the knee. (c, d) After the joint capsule was opened, the patella was dislocated laterally and exposed femoral condyles. (e) A corneal trephine with a diameter of 5 mm was used to outline the cartilage defect site. (f) Wound closure.

To establish a cartilage defect, rabbits were anesthetized with an intravenous injection of sodium pentobarbital (20 mg/kg). After general disinfection of the knee, an incision was made on the medial joint of the knee. After the joint capsule was opened, the patella was dislocated laterally and exposed femoral condyles. A corneal trephine with a diameter of 5 mm was used to outline the cartilage defect site. Noncalcified cartilage was scraped away using loupe visualization. The objective of modeling was to remove cartilage as much as possible without damaging the subchondral bone (Figure 1). After surgery, animals received antibiotics (penicillin for three consecutive days) and analgesics (buprenorphine for two days). Rabbits were monitored for the signs of activity, joint movement, local infection, and other complications.

Three weeks after surgery, rabbits were randomly divided into four groups for later injecting different therapeutic substances. We chose the lower and lateral edge of the patella for the injection of the substances and removed joint fluid by suction to confirm an accurate injection point. The following four groups were established: (i) group A (hUSCs plus HA, *n* = 6), 1 × 10^7^ cells and 1 mL 1% HA (pH 6.7, 1000 kDa, Furuida, China) were injected into the knee joint cavity; (ii) group B (hUSCs, *n* = 6), 1 × 10^7^ cells and 1 mL of normal saline were injected into knee joints; (iii) group C (HA group, *n* = 6), only 1 mL 1% HA was injected into knee joints; and (iv) group D is the control group with normal saline injected (*n* = 6).

#### 2.5.4. Gross Appearance

Twelve weeks after injection, 24 rabbits were sacrificed and 48 knees were harvested. Surrounding soft tissues were removed, and defective cartilage tissue was obtained. Afterward, two investigators evaluated the gross appearance of the cartilage tissue, including the degree of repair, integration to the border zone, and macroscopic appearance on the surface.

#### 2.5.5. Histological Analysis

Samples were washed twice with PBS, fixed in 4.0% paraformaldehyde for 7 days at 25–30°C, and decalcified in 10% formic acid for 3 months. After decalcification, the femoral condyles were cut into three pieces from lateral to medial condyle along the sagittal plane. All samples were embedded in paraffin and cut into 5 *μ*m thick sections that were stained with hematoxylin-eosin (HE), Masson, and toluidine blue. The cell morphology, color of the matrix, intactness of the surface, and thickness and integration of cartilage with adjacent host cartilage were evaluated.

#### 2.5.6. Immunohistochemical Analysis

Paraffin-embedded tissues were dewaxed with xylene, and endogenous peroxidase was blocked using 3% hydrogen peroxide. Sections were rinsed with PBS and blocked with goat serum (Sichuan University Ltd., China), followed by incubation with the primary antibodies: mouse anti-type II collagen and mouse anti-aggrecan (Novus, USA) for 12 h at 4°C. After washing three times for 5 min with PBS, sections were incubated with goat anti-mouse IgG (H + L) secondary antibodies (Peroxidase Affinipure, 115-035-003, Jackson, USA) for 30 min at room temperature as well as with peroxidase-conjugated streptavidin (Sichuan University Ltd., China). Next, sections were washed three times with PBS for 5 min. Finally, 3,3-diaminobenzidine (DAB) solution containing 0.01% hydrogen peroxide was added, and sections were counterstained with hematoxylin.

#### 2.5.7. Histological Score

To quantify the differences of treatment results in histological and immunohistochemical staining, a histological score was given according to the method described by Pineda et al. [17]. In brief, a scale from 0 (good) to 14 (severe) was given. In our study, filling of the defect, reconstitution of the osteochondral junction, matrix staining, and cell morphology were scored (Table 2).

### 2.6. Statistical Analysis

All values are expressed as the mean ± standard deviation (SD). Statistical analyses were performed using SPSS 17.0 software (SPSS, USA). Results were analyzed using Student's *t*-test, and *P* < 0.05 was considered statistically significant.

## 3. Results

### 3.1. Morphology and Characterization of hUSCs

**Figure 2:**
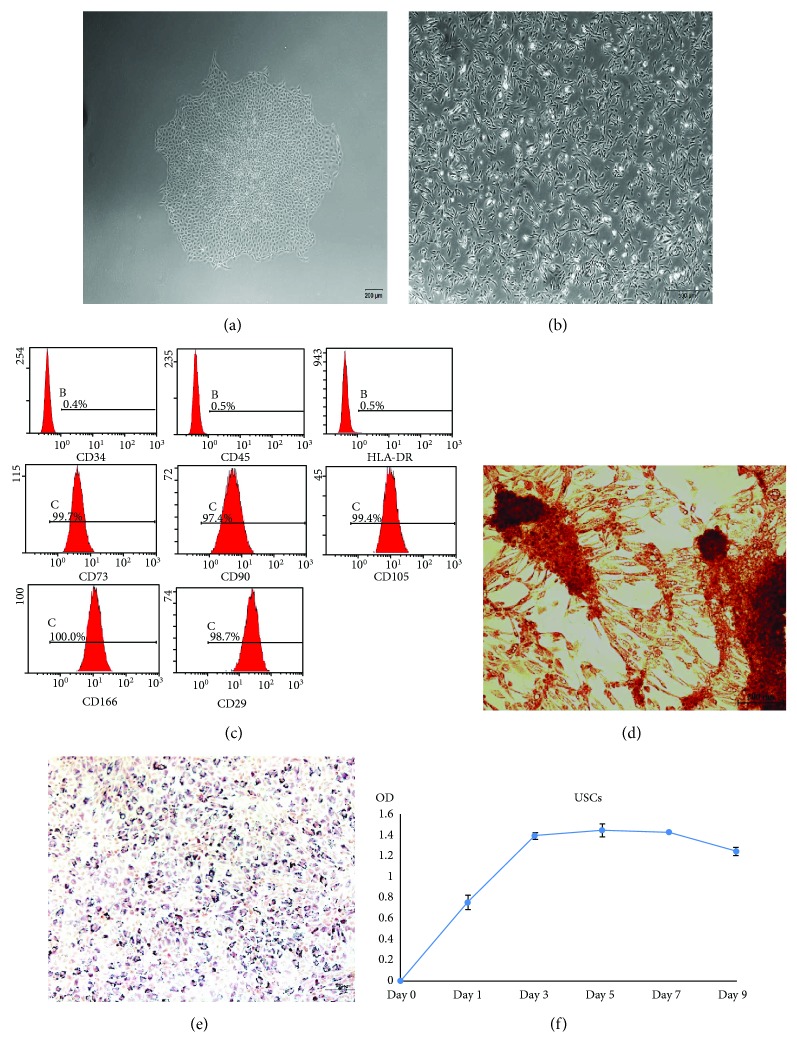
The morphology and characterization of hUSCs. (a) “Rice grain”-like appearance of hUSCs after initial plating. (b) Elongated morphology of hUSCs after several passages. (c) Flow cytometry results of hUSCs. (d) Osteogenic differentiation of hUSCs with Alizarin Red. (e) Adipogenic differentiation of hUSCs with Oil Red O. (f) Growth curves of hUSCs.

Cell colonies of hUSCs were observed 7–10 days after initial plating, in which the cells had “rice grain”-like appearance (Figure 2(a)). After several passages, hUSCs always exhibited an elongated morphology (Figure 2(b)). In addition, flow cytometry results showed that hUSCs had a positive staining for CD29, CD73, CD90, CD105, and CD166, but were negative for CD34, CD45, and MHC-II HLA-DR (Figure 2(c)). Figures 2(d) and 2(e) reveal that after culturing in specific induction media, hUSCs demonstrated to differentiate into an osteogenic or adipogenic lineage as indicated by the positive staining for Alizarin Red and Oil Red O, respectively. Moreover, the CCK-8 assay showed that the cells underwent a rapid growth phase from day 1 to day 3. After day 3, the growth slowed down (Figure 2(f)). The data indicated that cells isolated from human urine and maintained under specific culture conditions were classified as MSC.

### 3.2. *In Vitro* Chondrogenic Differentiation Potential of hUSCs

**Figure 3:**
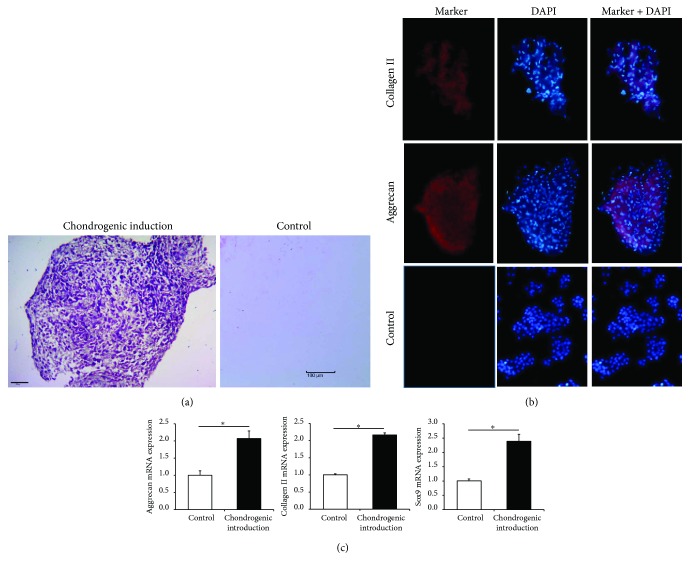
The chondrogenic differentiation potential of hUSCs *in vitro*. (a) Polysaccharides and proteoglycans were observed by toluidine blue staining after chondrogenic induction. (b) Immunofluorescence assay of chondrogenic-related markers (aggrecan and collagen II). (c) The mRNA expression of chondrogenesis-related genes (aggrecan, Sox9, and collagen II) was quantitated in hUSCs after 21 days of induction.

After 21 days of chondrogenic induction, toluidine blue staining of hUSCs indicated the presence of polysaccharides and proteoglycans (Figure 3(a)). The expression of chondrogenic-related markers, such as aggrecan and collagen II, was determined by immunofluorescence assay (Figure 3(b)). Furthermore, real-time PCR showed that the expression of chondrogenesis-related genes, aggrecan, Sox9, and collagen II was upregulated in induced hUSCs (Figure 3(c)).

### 3.3. Cell Morphology, Viability, and Proliferation in hUSCs-HA

**Figure 4:**
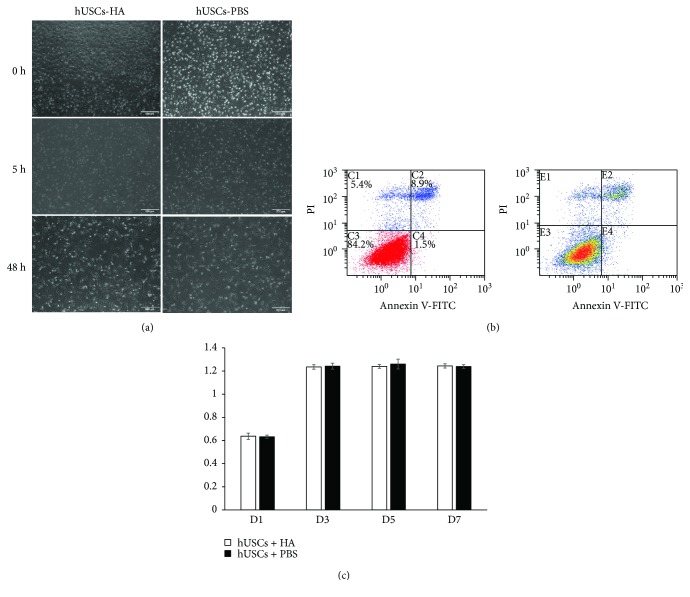
Cell morphology, viability, and proliferation about the hUSCs-HA system. (a) The comparison of morphology for hUSCs-HA and hUSCs-PBS at 0 h, 5 h, and 48 h. (b) Annexin V/PI assay of hUSCs seeded in HA. (c) Proliferation ability of cells in hUSCs-HA compared with those in hUSCs-PBS.

The morphology of hUSCs in HA was similar to hUSCs in PBS at 0 h, 5 h, and 48 h after the seeding (Figure 4(a)). The annexin V/PI assay demonstrated that after 24 h, 84.2% of hUSCs seeded in HA were still alive (Figure 4(b)). Besides, CCK-8 assay showed that the proliferation ability of the cells in the hUSCs-HA group was similar to that in the hUSCs-PBS group on days 0, 1, 3, 5, and 7 (Figure 4(c)).

### 3.4. Gross Appearance of Cartilage

**Figure 5:**
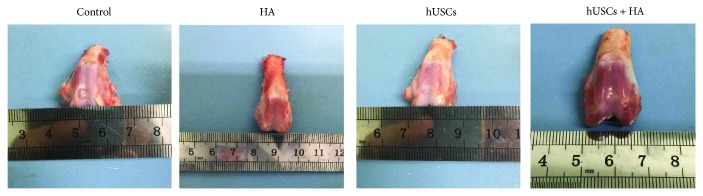
The gross appearance of the cartilage 12 weeks after injection.

Various degrees of cartilage damage were maintained 12 weeks after injection. The representative gross appearance of cartilage is shown in Figure 5. No significant change in degeneration was observed in knee joint cartilage, except for the defect in cartilage. For group A (hUSCs plus HA), newly formed cartilage-like tissue was frequently observed on the defect site, the surface color was relatively normal, and the newly formed cartilage-like tissue connected well with the surrounding cartilage tissue. Group B (hUSCs) also showed newly formed cartilage-like tissue. However, a scratch was noticed on the junction between the defect sites and normal sites. For group C (HA), some newly formed cartilage-like tissue was observed. An obvious scratch however was present on the junction between the defect sites and normal sites. In group D (the control group with normal saline injected), the cartilage defect was not recovered and newly formed cartilage-like tissue was hardly observed.

### 3.5. Histological Assessment of New Cartilage Formation in an *In Vivo* Cartilage Defect Model

**Figure 6:**
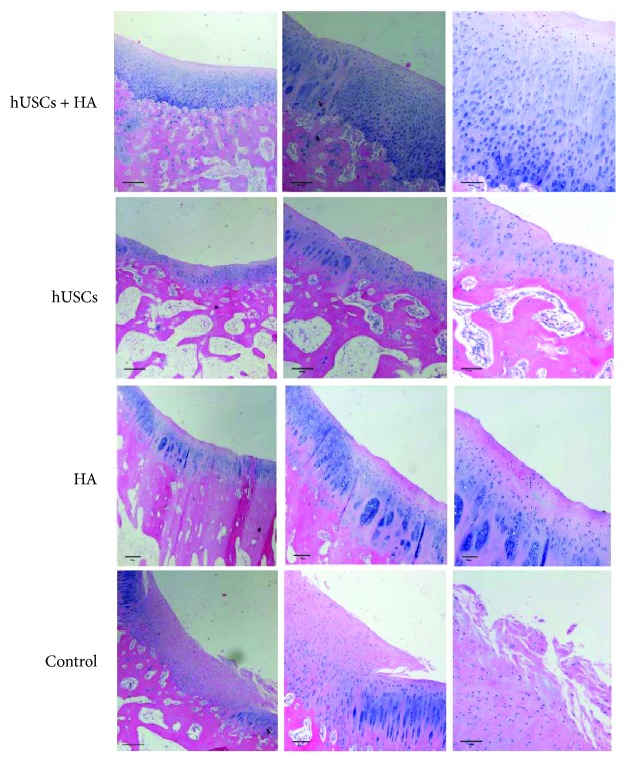
The HE staining of the cartilage 12 weeks after injection (scale bar = 500 *μ*m, 200 *μ*m, and 100 *μ*m).

HE staining, Masson staining, and toluidine blue staining were performed. Representative images of HE staining of newly formed cartilage in groups A, B, C, and D are shown in Figure 6. In group A, the defect site was covered by tissues similar to neocartilage, in which chondrocytes were present. The matrix staining had a normal appearance. In group B, sign tissues similar to cartilage and fibrous tissue were observed. In contrast, neocartilage-like tissue was seldom seen on the defect sites in groups C and D.

**Figure 7:**
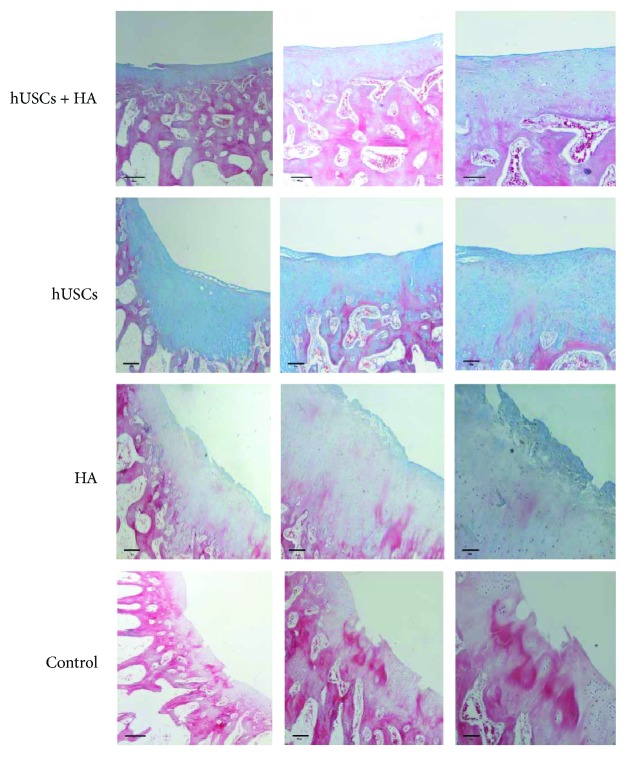
The Masson staining of the cartilage 12 weeks after injection (scale bar = 500 *μ*m, 200 *μ*m, and 100 *μ*m).

Representative images of Masson staining are shown in Figure 7. In group A, many chondrocytes were present. Tissues similar to cartilage fibers were observed regularly, and the color of the matrix was close to that in normal cartilage tissue. In group B, chondrocytes were hardly observed, while most cells were nonchondrocytes. The color of the matrix was pale when compared to that of normal cartilage. In groups C and D, no cartilage was observed at defective sites.

**Figure 8:**
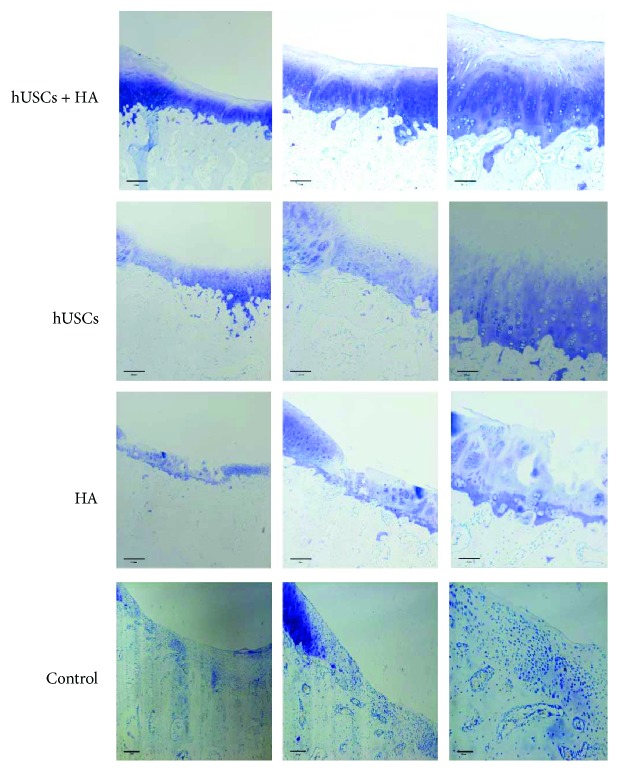
The toluidine blue staining of the cartilage 12 weeks after injection (scale bar = 500 *μ*m, 200 *μ*m, and 100 *μ*m).

Representative images of toluidine blue staining are shown in Figure 8. The staining in group A showed a darker blue staining at defect sites with a uniform layer of cartilage cells and clear tidemarks. In contrast, only a pale blue staining, few cartilage cells, a mass of fibrotic cells, and fiber tissue were observed in group B. No neocartilage tissue was observed in groups C and D.

**Figure 9:**
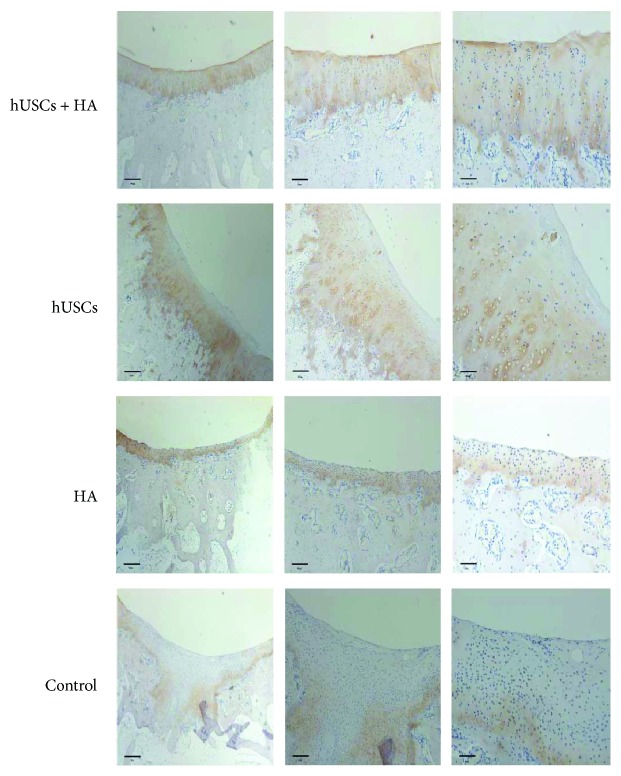
The type II collagen staining of the cartilage 12 weeks after injection (scale bar = 500 *μ*m, 200 *μ*m, and 100 *μ*m).

**Figure 10:**
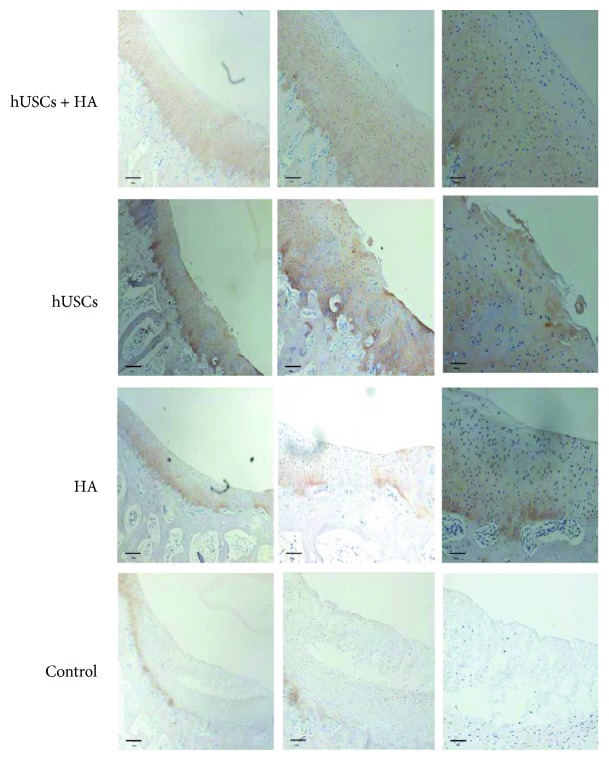
The aggrecan protein staining of the cartilage 12 weeks after injection (scale bar = 500 *μ*m, 200 *μ*m, and 100 *μ*m).

Representative images of the immunohistochemical analysis of the neocartilage from all four groups are presented in Figures 9 and 10. Group A exhibited a large number of chondrocyte cells. Moreover, the color of the defect sites in group A was similar to that of the surrounding tissue, which indicated that a significant amount of type II collagen and aggrecan protein was secreted. In group B, few cells similar to chondrocytes were observed and the color was light at the defect sites. In groups C and D, no newly formed tissue was detected. The data indicated the presence of increased collagen fibers and aggrecan protein in group A compared to groups B, C, and D. The histochemical analysis and immunohistochemistry implied that hUSCs in combination with HA stimulated the regeneration of cartilage more effectively than hUSCs alone or HA alone.

### 3.6. Histological Score

**Figure 11:**
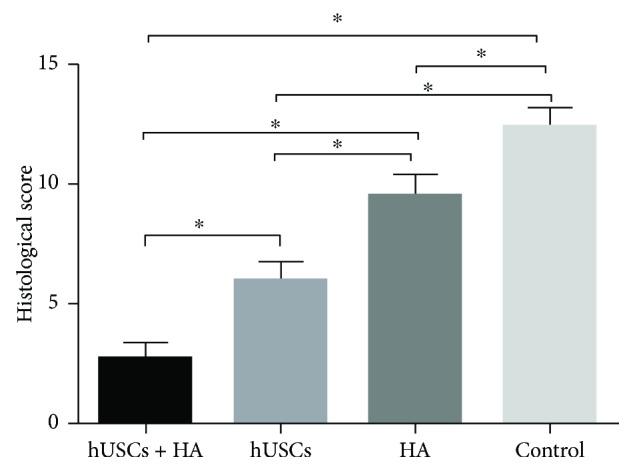
The histological score assessment 12 weeks after injection (^∗^*P* < 0.05).

The histological score for group A (2.75 ± 0.62) was significantly higher than the scores of group B (6 ± 0.74, *P* < 0.001), group C (9.58 ± 0.79, *P* < 0.001), and group D (12.41 ± 0.79, *P* < 0.001). These findings suggested that hUSCs-HA rather than hUSCs alone and HA alone was the most effective treatment in promoting the formation of neocartilage (Figure 11).

## 4. Discussion

In our study, we explored the cellular properties of hUSCs and compared their chondrogenic potency to differentiate into chondrocytes. After injection into cartilage defect knee joints in rabbits with or without HA, the deposition of aggrecan and collagen II was studied as a characteristic for neocartilage formation *in vivo*. The ability of hUSCs to self-renew and their differentiation potential were examined *in vitro*, whereas their ability to support novel cartilage formation in the cartilage defect model was assessed through histological assessments. These findings suggested that hUSCs could be a potential alternative therapeutic cell source for cartilage tissue engineering, especially when combined with HA.

Previous studies reported that stem cells appropriately seeded onto biomaterials could promote the regeneration of cartilage at defective sites [13, 18]. MSCs could be derived from a variety of human tissues, including bone marrow, skeletal muscle, adipose tissue, cord blood, skin, dental pulp, and endometrium, which have been reported in numerous studies previously [19–22]. The viable cell types with MSC characteristics in urine have recently been discovered [8, 10, 23]. In our study, we confirmed that hUSCs, when cultured under appropriate conditions as described above, possessed the properties belonging to MSCs. Although Pei et al. [24] reported that hUSCs did not show the ability to differentiate into chondrocytes in a 5% O_2_ and 5% CO_2_ incubator up to 14 days, Guan et al. [9], Bharadwaj et al. [10], and Gao et al. [25] have shown that hUSCs could differentiate toward the chondrogenic lineages after chondrogenic induction for 28 days. Kang et al. [26] reported that hUSCs could differentiate into chondrocytes but showed relatively lower chondrogenic differentiation rate compared to hASCs. Guan et al. [9] also demonstrated that hUSCs possessed biological characteristics similar to hASCs and had multilineage differentiation potential. Here, we successfully isolated hUSCs from human urine samples and demonstrated the capacity of hUSCs to differentiate into chondrocytes in a 20% O_2_ and 5% CO_2_ incubator for 21 days, based on the evidence of cell morphology, protein expression, and chondrogenesis-related gene expression. Ample evidence has validated that three-dimensional cultures are better than a monolayer culture for stabilizing the chondrocyte phenotype *in vitro* [27], which matches the method that we used to successfully induce hUSCs to differentiate into chondrocytes. Therefore, the culture environment and the incubation time may influence the result of chondrogenic induction of hUSCs. In addition, when compared with the autologous cell transplantation that relied on expensive and invasive surgery, our study revealed that hUSCs could potentially provide a low-cost and harmless way to cure cartilage defects caused by trauma injury and OA.

As reported by Venable et al. [28], HA may be associated with cartilage damage in early pathologic changes of OA. The treatment of OA by intra-articular HA injection has been commonly used to promote cartilage repair, and the mechanisms contributing to this function are proposed to include the suppression of proinflammatory cytokines and chemokines, the promotion of the anabolism, and the relief of pain [29, 30]. Nevertheless, the effects are controversial [31–33]. The discrepancy may be due to improper position of injection [34], suboptimal dosage of HA, or a prolonged interval between induction of OA and injection of HA. In this study, we injected HA, hUSCs, or a mixture thereof into knee joints of rabbits with cartilage defects and compared the recovery levels of articular cartilage. When compared with other groups, cartilage regeneration in the hUSCs + HA group was found as the best and the corresponding histological score was the highest. Besides, in the hUSCs + HA group, neocartilage-like tissue covered the defect site, chondrocytes were found in the recovered tissue, and matrix staining was normal. In contrast, only a few neocartilage-like tissues were found in other groups. Our results are in line with the findings of several earlier studies. Neocartilage was also reported by Grigolo et al., who demonstrated that after 24 weeks of treatment with chondrocytes and HA derivatives, cartilage defects in the rabbit knee were repaired [18]. Researchers in South Korea demonstrated positive treatment effects when using a similar approach [35]. A possible explanation may be that chondrocytes seeded onto appropriately configured synthetic biodegradable polymers adhere and perform different functions *in vitro*, as demonstrated by matrix formation [18]. Surgical implantation of the hUSCs + HA cell-polymer mixture in animals resulted in the formation of new cartilage that matures over time and showed the expression of collagen type II and synthesized proteoglycans [18].

Although the findings of this study are promising, several limitations need to be addressed before clinical applications and further studies are warranted to reveal: (1) how to improve chondrogenic capacity of hUSCs because the expression levels of all chondrogenic genes were relatively low [24, 26]; (2) the advantages of hUSCs over other MSCs by comparing the chondrogenic ability of hUSCs with those of other MSC types, such as hBMSCs and hASCs; and (3) the molecular mechanisms involved in the interactions between hUSCs and HA.

Based on the results of our previous study and the measured values of the pH, osmotic pressure, and the survival rate of stem cells in solution, we chose 1% HA physiological saline solution as our intervention means. The ability of cell proliferation was tested using CCK-8 assay, which showed that the proliferation ability of the cells in the hUSCs-HA group was similar to that of the cells in the hUSCs-PBS groups at days 0, 1, 3, 5, and 7. Noticeably, the HA physiological saline solution only showed minor side effects on hUSC activity. In a study by Shapiro et al. [36], it was implied that cartilage defects in rabbits could be repaired by the animal itself in 6 weeks. But it could be achieved only if the diameter of the defect is smaller than 3 mm, the depth of the defect touched the subchondral bone, and intervention is absent. To ensure the reliability of our experiments and solve the doubt about self-healing, we expanded the diameter of the defect to 5 mm and removed all cartilage without damaging the subchondral bone to avoid any interference from bone marrow-derived MSCs.

## 5. Conclusion

In summary, the findings of our study indicated that, according to the stem cell characteristics, hUSCs can be classified into the MSC family. hUSCs are able to differentiate into chondrocytes with characteristic deposition of aggrecan and collagen II *in vitro*. Furthermore, hUSCs-HA can stimulate significantly more neocartilage formation compared with hUSCs, HA, and saline. These results, along with the findings of our previous study, indicated that hUSCs could be an alternative therapeutic cell source for cartilage tissue engineering and a promising candidate for, especially when combined with HA.

## Figures and Tables

**Table 1:** Primer sequences (5′-3′) used for RT-PCR.

Gene	Primer sequences (5′ → 3′)	Amplification size (bp)
hCOL2A1F	GCTCCCAGAACATCACCTACC	192 bp
hCOL2A1R	CAGTCTTGCCCCACTTACCG	
hSox9F	CTCCTACCCGCCCATCAC	114 bp
hSox9R	TAGGTGAAGGTGGAGTAGAGGC	
hACANF	GCCTATCAGGACAAGGTCTCAC	185 bp
hACANR	ATGGCTCTGTAATGGAACACGA	
h-Actin r	CTGGAAGGTGGACAGCGAGG	205 bp
h-Actin f	TGACGTGGACATCCGCAAAG	

**Table 2:** The semiquantitative scale for grading the natural healing articular cartilage: subcategories and their individual respective scores.

Feature	Score
Filling of defect	
100%	0
75%	1
50%	2
25%	3
0%	4
Reconstitution of osteochondral junction	
Yes	0
Almost	1
Not close	2
Matrix staining	
Normal	0
Reduced staining	1
Significantly reduced staining	2
Faint staining	3
No stain	4
Cell morphology	
Normal	0
Mostly hyaline and fibrocartilage	1
Mostly fibrocartilage	2
Some fibrocartilage but mostly nonchondrocytic cell	3
Nonchondrocytic cell only	4
